# Numerical Signature Dataset of Curculionidae and Tenebrionidae Beetle Fragments for ML Identification

**DOI:** 10.1038/s41597-025-06309-6

**Published:** 2025-12-12

**Authors:** Ronnie O. Serfa Juan, Alison R. Gerken

**Affiliations:** USDA ARS, Center for Grain and Animal Health Research, Manhattan, KS USA

**Keywords:** Agriculture, Research data

## Abstract

This data descriptor presents a curated dataset of numerical signature descriptors derived from fragment images of six economically significant stored-product beetle species from the families Curculionidae (*Sitophilus zeamais*, *Sitophilus oryzae*, *Sitophilus granarius*) and Tenebrionidae (*Tribolium castaneum*, *Tribolium confusum*, *Latheticus oryzae*). Anatomical fragments—including antennae, elytra, thorax, snout (Curculionidae), and head aspect ratio (Tenebrionidae)—were imaged using digital microscopy and processed with standardized image acquisition and segmentation techniques. From each image, four statistical descriptors—skewness, kurtosis, entropy, and standard deviation—were extracted, which form compact numerical signatures that capture fragment-level texture and morphological variation. These descriptors are designed to support artificial intelligence and machine learning workflows for automated classification in entomological diagnostics and post-harvest pest detection. The dataset includes 3,423 fragment images, each linked to a numerical signature vector and labeled by species, anatomical region, and metadata. This dataset adheres to Findable, Accessible, Interoperable, Reusable (FAIR) principles and is intended for open reuse in entomological AI research and machine learning-driven insect fragment identification workflows.

## Introduction

Stored product insects, such as species within the Curculionidae and Tenebrionidae families, are among the most significant global pests affecting post-harvest grain and food commodities. They contribute to substantial economic losses through direct feeding damage, contamination, and reduced product quality in bulk storage and food distribution systems. Often, diagnostics relies on fragmented insect body parts recovered from grain samples are used as forensic indicators of infestation, with thresholds directly influencing regulatory decisions and trade acceptance^1–3^. Traditionally, the identification of insect fragments has been performed manually by trained entomologists using light microscopy and reference keys. However, this method is time-consuming, subjective, and increasingly challenged by the shortage of taxonomic expertise and the fragmentary nature of field samples^3,4^. As a result, there is growing interest in the application of image-based morphometric analysis and artificial intelligence (AI) to automate and standardize fragment classification^4,5^.

In this dataset, we present fragment-level digital images and their corresponding numerical signature descriptors derived from six key stored-product beetle species. Anatomical regions—antennae, elytra, thorax, snout (for Curculionidae), and head aspect ratio (for Tenebrionidae)—were isolated and analyzed using image processing to extract four statistical features: skewness, kurtosis, entropy, and standard deviation. These descriptors were selected based on the extraction of low-level intensity and texture-based features from grayscale images, which are critical in quantifying surface morphology. Specifically:

- Skewness measures the asymmetry of intensity distribution, which helps identify directional patterns or light bias in the texture.
- Kurtosis characterizes the sharpness or flatness of the intensity profile, useful in detecting edge concentration or fine surface details.
- Entropy quantifies the randomness or complexity of pixel arrangements, reflecting surface irregularity and texture granularity.
- Standard deviation captures the spread of pixel intensity values, indicating contrast variation and textural roughness.

These numerical signatures provide a compact, machine-readable representation of each fragment, enabling differentiation between visually similar insect species even when only partial anatomical segments are available for analysis. Our aim is to enable entomologists, data scientists, and agricultural practitioners to leverage these machine-readable morphometric features in artificial intelligence and machine learning workflows for automated classification and pest detection. The dataset supports the development of intelligent post-harvest inspection systems and aligns with global efforts to promote data-driven solutions for food security, pest management, and sustainable storage practices^6,7^.

This dataset represents one of the first publicly available resources to offer standardized morphometric descriptors specifically derived from insect fragment images, with a design framework explicitly tailored for downstream integration into AI and machine learning classification systems. It extends previous efforts focused on whole-insect identification by addressing the practical challenges of fragment-based diagnostics, particularly in operational settings such as post-harvest storage facilities and grain inspection systems where complete specimens are rarely available.

## Methods

**Fig. 1:**
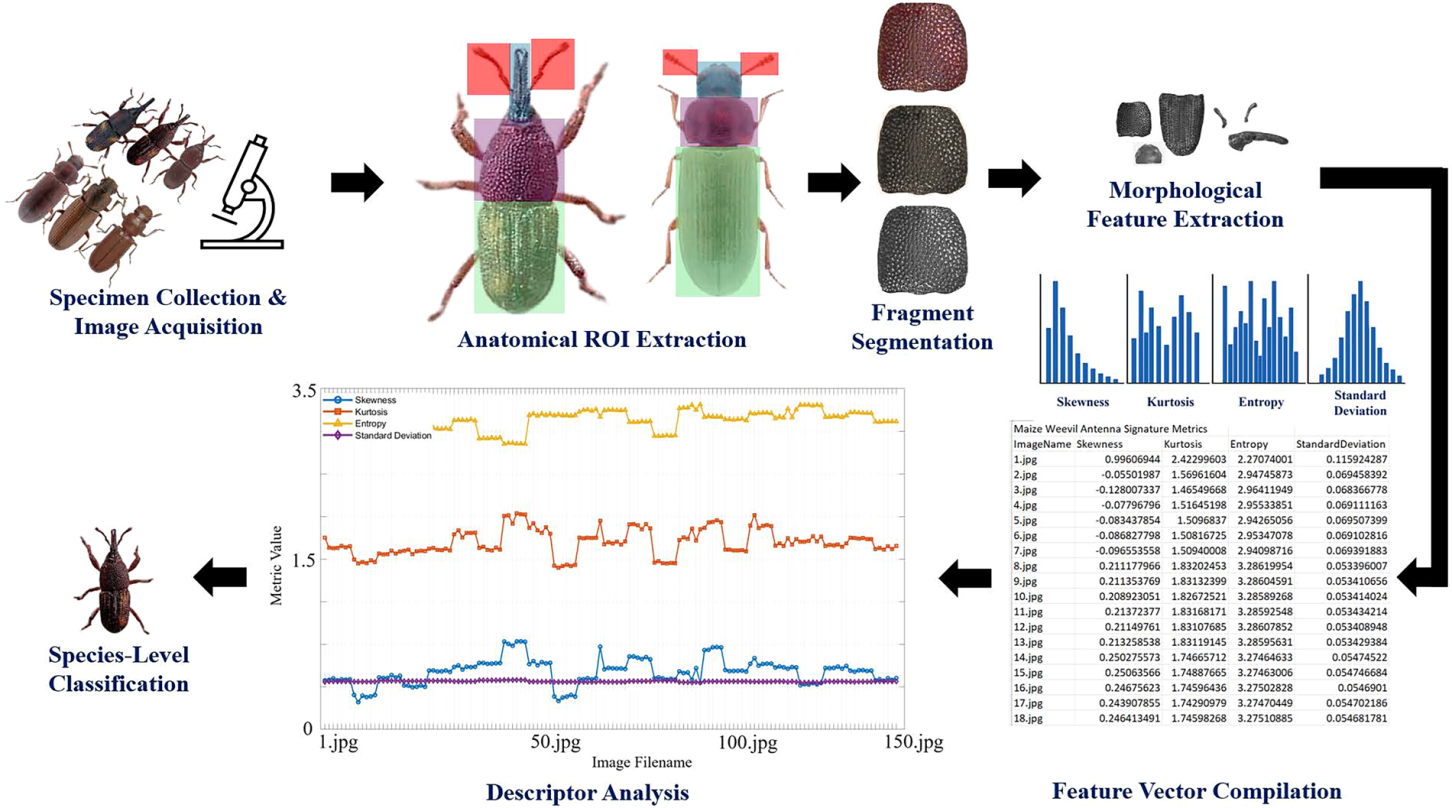
Methodological Workflow for Fragment-Based Numerical Profiling of Stored Product Beetles.

The complete methodological pipeline is illustrated in Fig. 1, which outlines the steps from image acquisition to feature extraction and signature analysis for insect classification.

### Specimen collection and fragmentation

**Fig. 2:**
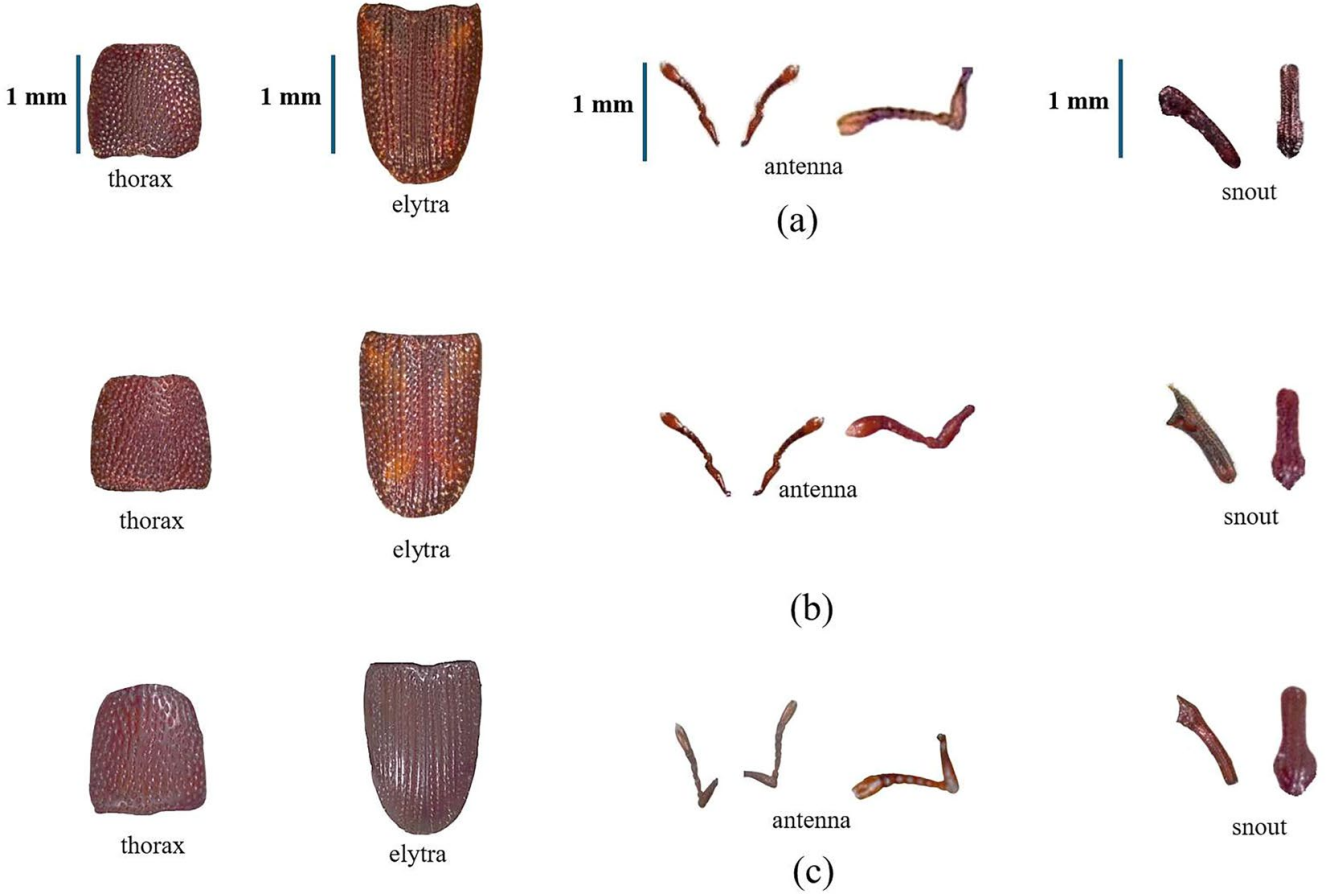
Representative anatomical fragments of the Curculionidae triad beetle species used for dataset construction. Row (**a**) *S. zeamais*, (**b**) *S. oryzae*, and (**c**) *S. granarius* are represented by their thorax, elytra, antenna, and snout fragments.

**Fig. 3:**
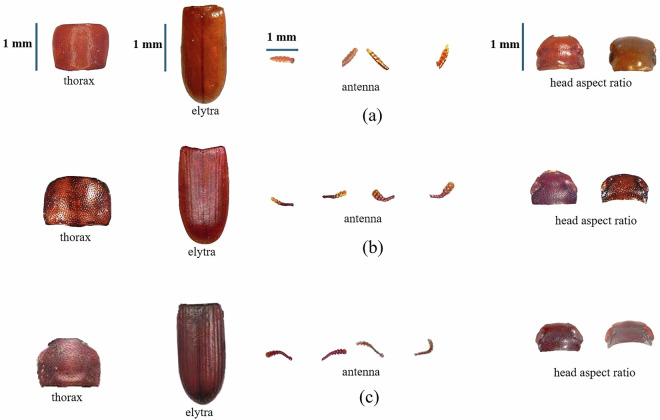
Representative anatomical fragments of the Tenebrionidae triad beetle species used for dataset construction. Row (**a**) *L. oryzae*, (**b**) *T. castaneum*, and (**c**) *T. confusum* are represented by their thorax, elytra, antenna, and head aspect ratio fragments.

Representative specimens from six stored-product beetle species were selected from curated entomological collections. These included three members of the Curculionidae family—*Sitophilus zeamais*, *Sitophilus oryzae*, and *Sitophilus granarius*—and three from the Tenebrionidae family—*Tribolium castaneum*, *Tribolium confusum*, and *Latheticus oryzae*. Figures 2 and 3 show representative fragment-level images of these six beetle species. The *S. zeamais* (maize weevil), *S. oryzae* (rice weevil), and *S. granarius* (wheat weevil) from the family Curculionidae are shown with anatomical fragments including elytra, antennae, thorax, and snout. The *L. oryzae* (long-headed flour beetle), T. *castaneum* (red flour beetle), and T. *confusum* (confused flour beetle) from the family Tenebrionidae are represented by elytral segments, thoracic sections, and head aspect ratios. These fragment images illustrate the morphological variability inherent across species and anatomical regions, supporting the feasibility of utilizing partial insect remains for diagnostic and classification tasks. The structural characteristics of each fragment were captured through standardized digital imaging and subsequently quantified using statistical descriptors—specifically skewness, kurtosis, entropy, and standard deviation. This visualization underscores the foundation of fragment-based morphometric analysis as a robust and interpretable input for machine learning classification, benchmarking, and the development of AI-powered entomological decision-support systems.

### Image acquisition

Fragment images were acquired using the Region of Interest (ROI) extraction function in MATLAB. Anatomical fragments—including antennae, elytra, thorax, snout, and head—were precisely segmented from whole-insect images using an ROI-based isolation workflow. Specimens were positioned against a uniform white background to minimize reflectance and suppress optical noise during imaging. Each fragment was exported at a standardized resolution of 256 × 256 pixels in JPEG format, selected to balance morphological detail preservation with computational efficiency, and to ensure compatibility with common convolutional neural network (CNN) architectures used in image-based classification. To maintain consistency, all images were spatially aligned and scaled using calibration slide references, followed by automated normalization to standardize orientation and magnification. The complete methodological setup for the image acquisition process, including the use of a handheld digital microscope, is detailed in the Supplementary Materials.

### Metadata consistency and data curation

To ensure the integrity and traceability of each data sample, a consistent metadata tagging and file naming system was implemented. Every image fragment was assigned a unique identifier composed of species name, and anatomical region. This convention enables traceable linking between the raw input image, the extracted Region of Interest (ROI), the processed grayscale version, and the resulting numerical signature file. Metadata—including species, anatomical region, augmentation type, and capture resolution—was logged during the annotation phase using an interactive MATLAB prompt to reduce human error and enforce standardized labeling.

Consistency across metadata entries was further validated using script-based checks for naming conformity and missing fields. In cases where ambiguity or visual artifacts were observed (e.g., low resolution, fragment overlap, or occlusion), such images were excluded from the dataset.

### Image preprocessing

To ensure consistency and reliability in feature extraction, all images were subjected to a standardized preprocessing pipeline. This included grayscale conversion to simplify intensity analysis, histogram equalization to enhance contrast, binary thresholding and segmentation to isolate anatomical fragments from the background, and noise reduction to suppress irrelevant artifacts. These operations improve image uniformity and emphasize structural features critical to morphometric analysis. Preprocessed images were reviewed to confirm anatomical accuracy, alignment, and integrity—ensuring that each fragment maintained a clear and consistent representation for subsequent descriptor extraction^8,9^.

### Numerical signature extraction

From each preprocessed grayscale image, four statistical descriptors—skewness, kurtosis, entropy, and standard deviation—were extracted using MATLAB R2024b. These parameters were computed using built-in functions: entropy, std2, skewness, and kurtosis, applied to the grayscale pixel intensity values. This process transforms visual information into standardized numerical representations that quantify intensity distribution, texture complexity, and structural symmetry. The resulting signature vectors provide robust and reproducible input features for machine learning models, enabling reliable classification of insect fragments. These parameters were extracted in MATLAB using the following functions:

grayImage = rgb2gray(inputImage);

stdVal = std2(grayImage);

entropyVal = entropy(grayImage);

skewVal = skewness(double(grayImage(:)));

kurtVal = kurtosis(double(grayImage(:)));

## Sample Numerical Signature Results and Discussion

**Fig. 4:**
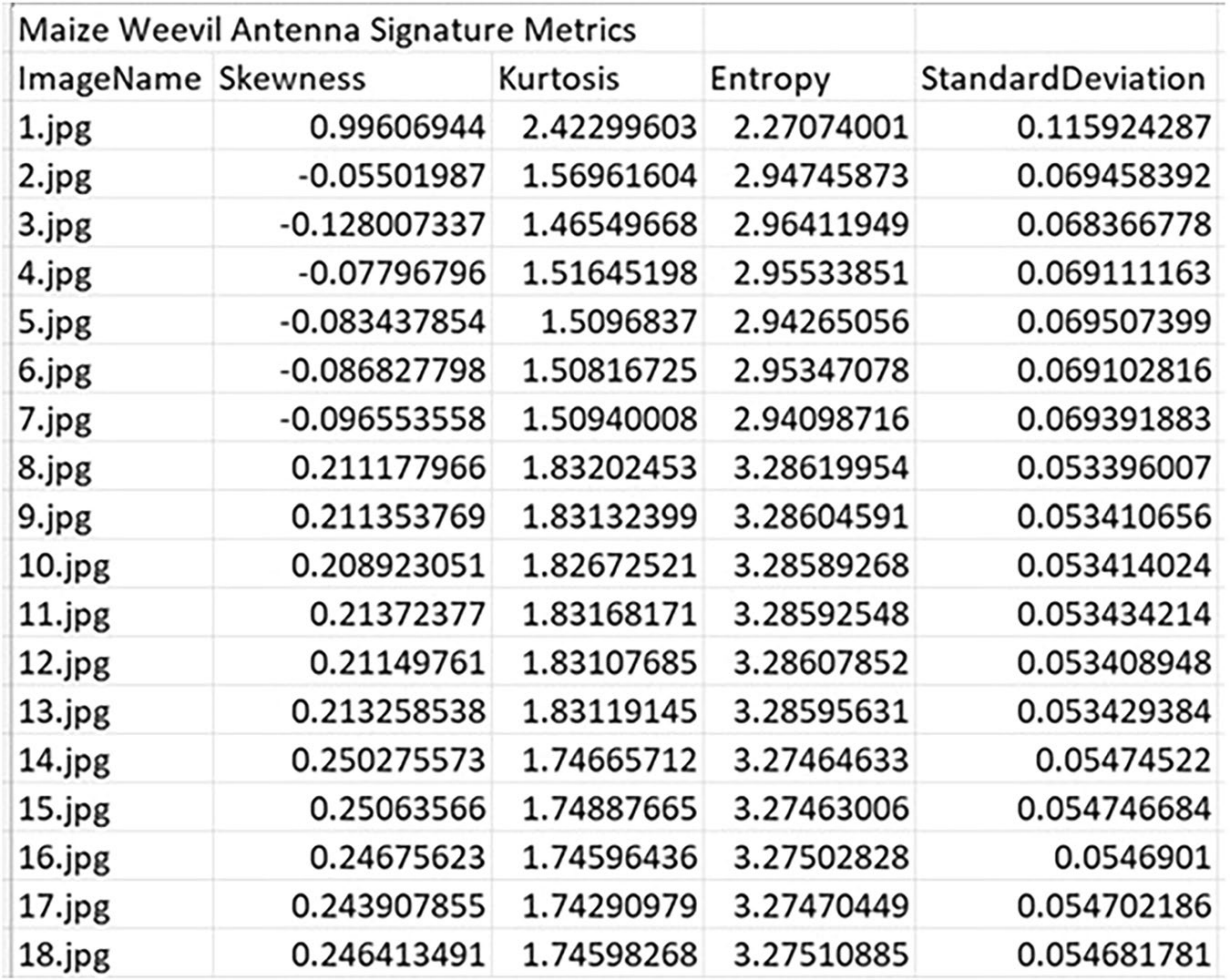
Sample numerical signature metrics of skewness, kurtosis, entropy, and standard deviation for *S. zeamais* antenna fragments.

**Fig. 5:**
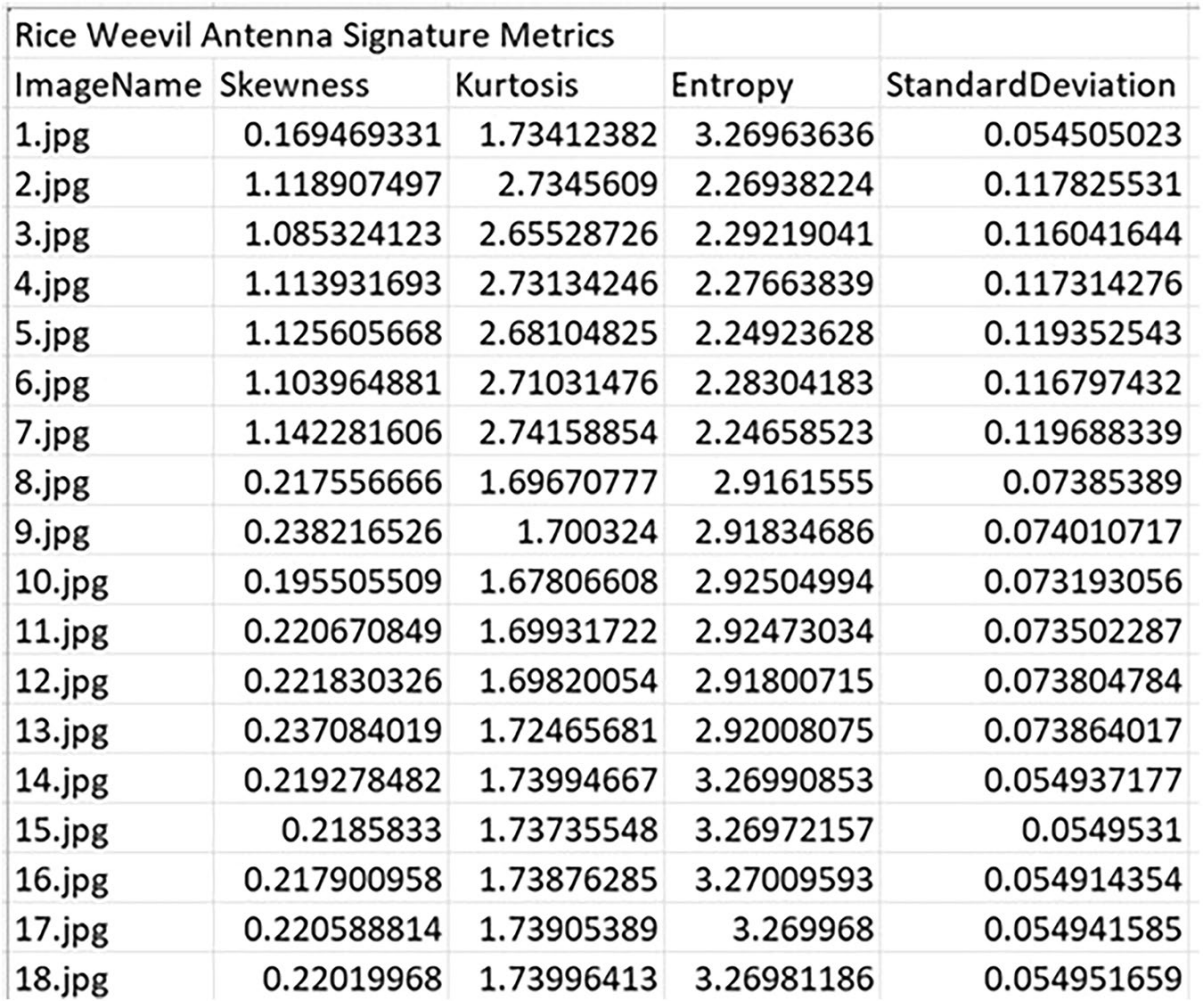
Sample numerical signature metrics of skewness, kurtosis, entropy, and standard deviation for *S. oryzae* antenna fragments. Descriptors reflect intensity and morphological variations useful for fragment-level classification.

**Fig. 6:**
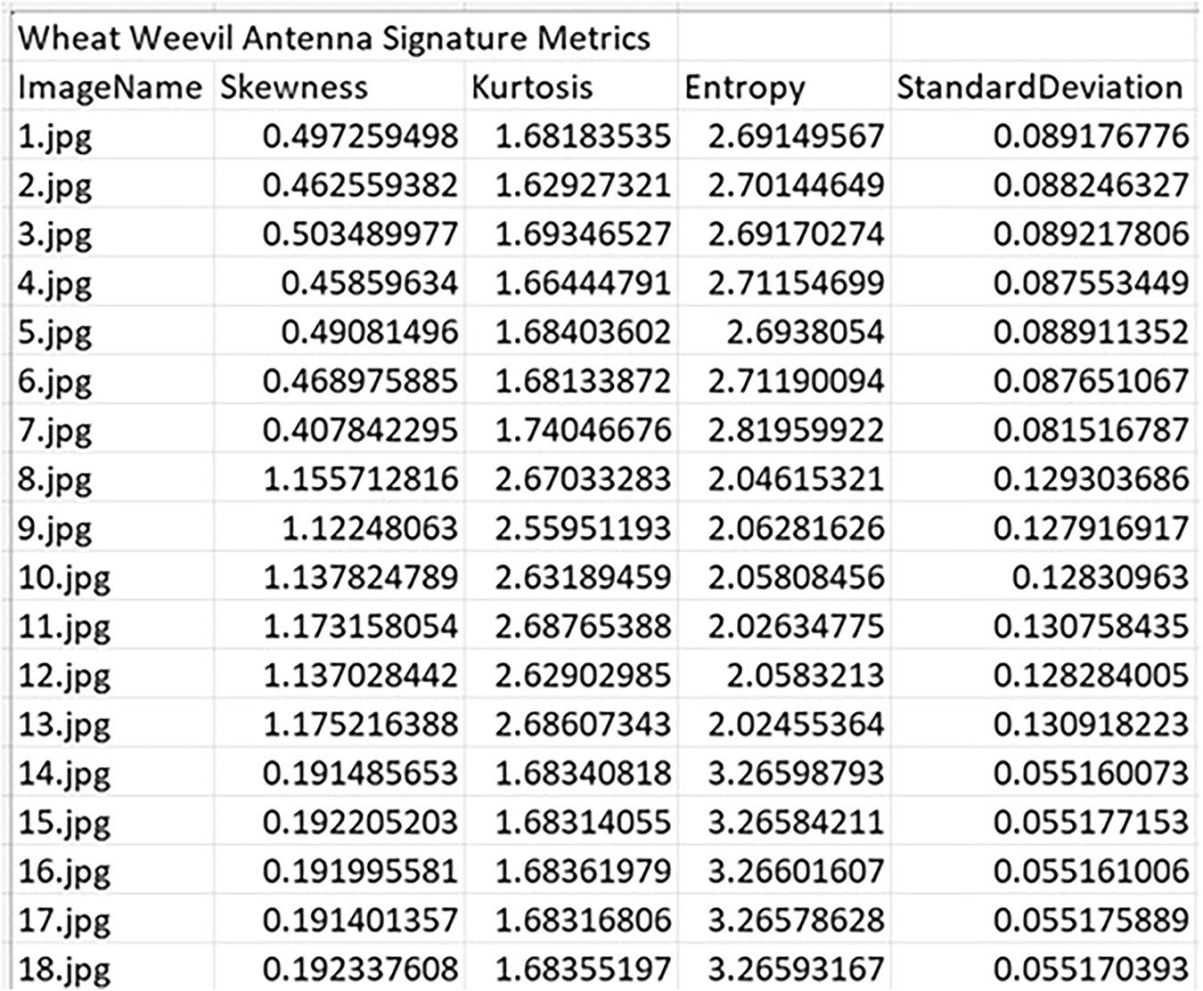
Sample numerical signature metrics of skewness, kurtosis, entropy, and standard deviation for *S. granarius* antenna fragments. The quantified values highlight measurable distinctions relevant to machine learning benchmarking tasks.

To evaluate the effectiveness of fragment-level morphological analysis, statistical descriptors were extracted from antenna images of three species: *S. zeamais*, *S. oryzae*, and *S. granarius*. The parameters were computed from grayscale pixel intensity values of each fragment image. These descriptors form the basis of the numerical signature for each sample, capturing measurable variation in shape and texture across species. Figures 4 to 6 present representative samples of the computed numerical signatures for antenna fragments across the three weevil species. The descriptors reveal intra- and inter-species variability in image complexity and intensity distribution, which can be used for downstream classification tasks. For instance, *S. oryzae* antenna fragments show consistently higher skewness and kurtosis compared to *S. zeamais* or *S. granarius*, suggesting species-specific texture characteristics preserved at the fragment level. The full set of numerical signature outputs—covering all anatomical fragments per species—is provided as Excel files and is publicly accessible via the dataset repository at the USDA-ARS Ag Data Commons. These files are located under the folder titled “Numerical Signatures”, organized by species and fragment type. The dataset supports benchmarking of machine learning models and reproducibility in future fragment-based entomological studies.

### Data labeling and organization

All data were labeled by family, species, and fragment type, and structured with unique IDs linking raw images, processed images, and numerical signature vectors. Metadata includes species name, image resolution, and capture details.

### Applications and use cases

Potential applications include integration with real-time sensors using embedded AI, the development of classifiers for grain inspection agencies, or benchmarking performance in academic research across different ML architectures. This dataset also opens the door for novel multi-modal research combining vision-based morphology with acoustic signatures, as demonstrated in recent insect-sound datasets^10^.

## Data Records

The dataset is publicly available on the USDA National Agricultural Library Ag Data Commons (10.15482/USDA.ADC/29066444 and Figshare 10.15482/USDA.ADC/29066444.v1) under an open-access CC0/CC BY license^11,12^. It comprises raw and processed beetle images, numerical signature descriptors, and comprehensive metadata. Each record links a unique image ID with species, family, anatomical fragment, resolution, and computed statistics (skewness, entropy, kurtosis, standard deviation). The metadata is organized in a well-structured CSV file to ensure transparency, reproducibility, and traceability, with clear associations between full-body images, extracted ROIs, and derived features.

Unlike traditional whole-insect datasets, this resource emphasizes anatomical fragments—addressing practical challenges such as damaged specimens in grain storage or forensic contexts. Aligned with FAIR principles, the dataset is findable via persistent DOI, accessible through open repositories, interoperable across workflows, and reusable in various AI pipelines. It is optimized as a benchmarking tool for fragment-based insect classification, supporting machine learning models including SVM, k-NN, random forests, and CNNs. Its lightweight numerical features enable rapid model inference, suitable for deployment in real-time, embedded applications such as automated pest detection in post-harvest monitoring systems.

By enabling early and accurate species-level identification from incomplete remains, this dataset supports smarter pest management strategies and contributes to agricultural sustainability and food security.

## Technical Validation

To ensure that this dataset is both reliable and practical for future research, we placed strong emphasis on validation at each stage of its development.

1. Metadata consistency. Every fragment was carefully tracked using unique identifiers that link the original image, its extracted Region of Interest (ROI), the preprocessed grayscale fragment, and the resulting numerical signature vector. Automated scripts were used to check for missing entries or formatting inconsistencies, which helped maintain a clean and standardized metadata framework.
2. Image quality and selection. Not all captured fragments were suitable for inclusion. Images that showed excessive noise, unclear anatomical boundaries, or overlapping fragments were excluded after careful inspection. This step ensured that only high-quality, representative fragments formed part of the dataset.
3. Preprocessing and normalization. The preprocessing pipeline—grayscale conversion, histogram equalization, thresholding, and noise reduction—was verified to make sure that anatomical integrity was preserved. Calibration slides were also used to confirm that scaling and orientation were consistent across samples, which is essential for downstream machine learning applications.
4. Labelling accuracy. Custom MATLAB scripts were developed to automatically cross-check species names, anatomical regions, and augmentation types against controlled vocabularies. This minimized human error during annotation and enforced standardized naming conventions. By combining automated checks with manual review, we built confidence in the accuracy, consistency, and reproducibility of the dataset. These validation steps ensure that the data are not only technically sound but also well-prepared for integration into machine learning workflows, entomological studies, and applied pest management research.

## Usage Notes

The dataset consists of laboratory-acquired images under controlled lighting; performance in field conditions may vary and would require additional training data or domain adaptation. Fragment visibility and image quality significantly influence descriptor values; robust preprocessing is recommended. Ethical and fair use of the dataset must ensure proper citation and adherence to licensing terms when used for commercial or academic model development. Future work may expand this dataset with multi-angle imagery, temporal sequences, or hyperspectral attributes to further increase classification robustness across environments and fragment conditions. One limitation of the current version of the dataset is the absence of inter-annotator variability or multi-annotator labeling, which could affect reproducibility under different user conditions. In addition, all fragment images were acquired in laboratory-controlled settings; no field-captured fragments were included in this version. This may limit the model’s generalization capabilities in uncontrolled or degraded real-world scenarios, particularly in low-light or damaged-fragment conditions.

## Data Availability

The dataset supporting this publication has been deposited in the USDA National Agricultural Library’s Ag Data Commons under the 10.15482/USDA.ADC/29066444 and is mirrored in Figshare under 10.15482/USDA.ADC/29066444.v1. Both repositories include raw and processed beetle fragment images, numerical signature descriptors, and associated metadata. All data are available under a CC0 / CC BY open license to support reuse and reproducibility.

## Code availability

All MATLAB scripts used for ROI extraction, augmentation, metadata logging, and numerical signature computation are available in a public GitHub repository: https://github.com/pece71/beetle-fragments-numerical-signatures. The repository includes versioned scripts compatible with MATLAB R2024b, a minimal test dataset, and a step-by-step usage guide with expected input/output examples.

Data license: CC0 / CC BY 4.0 (as applicable to the dataset DOIs).

Code license: MIT License. The repository includes a LICENSE file specifying open access use, reuse, and modification rights under the MIT License.

Per journal guidance, the full source code is not printed in the manuscript; readers can access, run, and reproduce the workflow directly from the repository.
